# The Dual Role of Dietary Phytochemicals in Oxidative Stress: Implications for Oncogenesis, Cancer Chemoprevention, and ncRNA Regulation

**DOI:** 10.3390/antiox14060620

**Published:** 2025-05-22

**Authors:** Khalid Umar Fakhri, Deepti Sharma, Homa Fatma, Durdana Yasin, Manzar Alam, Neha Sami, Farhan Jalees Ahmad, Anas Shamsi, Moshahid Alam Rizvi

**Affiliations:** 1 Genome Biology Lab, Department of Biosciences, Jamia Millia Islamia, New Delhi 110025, India; khalid148147@st.jmi.ac.in; 2 Amity Indian Military College for Women, Amity University, Noida Campus, Noida 201313, Uttar Pradesh, India; dsharma22@amity.edu; 3 Department of Zoology, Aligarh Muslim University, Aligarh 202002, Uttar Pradesh, India; hfatma@myamu.ac.in; 4 Department of Biosciences, Integral University, Lucknow 226026, Uttar Pradesh, India; durdana@iul.ac.in; 5 Centre for Interdisciplinary Research in Basic Sciences, Jamia Millia Islamia, New Delhi 110025, India; manzar987@gmail.com; 6 Cyanobacterial Biotechnology Lab, Department of Biotechnology, Jamia Millia Islamia, New Delhi 110025, India; gf.nsami@jmi.ac.in; 7 Department of Pharmaceutics, School of Pharmaceutical Education and Research, Jamia Hamdard, New Delhi 110062, India; fjahmad@jamiahamdard.ac.in; 8 Center for Medical and Bio-Allied Health Sciences Research, Ajman University, Ajman P.O. Box 346, United Arab Emirates

**Keywords:** antioxidant, oxidative stress, ncRNA, phytochemicals, reactive oxygen species

## Abstract

Oxidative stress (OS), resulting from an imbalance between reactive oxygen species (ROS) and the antioxidant defense system, plays a critical role in the initiation and progression of cancer. Recent research has highlighted the regulatory influence of non-coding RNAs (ncRNAs) on cancer initiation and development through the regulation of redox homeostasis and key signaling pathways, which makes them potential targets for anticancer therapy. The ncRNA–oxidative stress axis contributes to malignancy through DNA damage, altered signaling, and dysregulated molecular networks. Plant-derived dietary components and phytochemicals have garnered significant attention for their ability to restore ROS balance and modulate the ncRNA/OS axis, thereby inhibiting carcinogenesis and enhancing the efficacy of chemotherapy. To study the interplay between OS, ncRNAs, and the anticancer potential of plant-derived compounds, in this review, we conducted an extensive search of electronic databases to identify and analyze studies that explore the interwork between OS, ncRNAs, and the chemotherapeutic role of phytochemicals. We discuss the dual role of phytochemicals in both cancer induction and suppression, emphasizing their capacity to generate ROS and regulate ncRNA expression. Furthermore, the review underscores the importance of nutritional interventions and antioxidant regulation in cancer chemoprevention and therapy, including the interconnected roles of oxidative stress, ncRNAs, and phytochemicals in cancer development and treatment, with a focus on dietary modulation as a strategic approach in oncology.

## 1. Introduction

Cancer is a complex condition characterized by the uncontrolled proliferation of dysregulated cells, resulting in a local and distant invasion of other body parts. In 2022, cancer was one of the primary causes of death at the global level, with an estimated 20 million new incidences and around 10 million deaths [1]. Cancer incidence is driven by both genetic and environmental factors, with most cases arising from lifestyle and environmental influences. While 5–10% of cancers are linked to inherited genetic mutations, the majority (90–95%) result from acquired genetic abnormalities caused by endogenous/exogenous or environmental factors such as pollutants, tobacco, infectious agents, lifestyle, and diet, which further alter cellular processes by altering tumor suppressor genes and proto-oncogenes [2]. By 2050, 6.9 million adults, 80 years or older, are expected to be diagnosed with cancer globally (20.5% of all cancer cases), and the number of deaths is expected to increase by double in the US alone [3,4]. Therefore, effective chemo-preventive strategies must be developed when considering human life expectancy and environmental conditions.

Under normal conditions, a balance between ROS generation and the activity of detoxification enzymes safeguards critical macromolecules including mitochondrial and chromosomal DNA, the proteome, and the transcriptome thereby maintaining effective defenses against cancer development [5]. Stressors such as oxidizing agents or disrupted metabolic pathways lead to excessive production of reactive oxygen species (ROS) leading to acquired genetic mutations which further results in cancer initiation [6]. Further, non-coding RNAs (ncRNAs) regulate oxidative stress in cancer by modulating antioxidant defenses (Nuclear factor erythroid 2-related factor 2 (Nrf2)/ Kelch-like ECH-associated protein 1 (KEAP1) and Sirtuin 1 (SIRT1) pathways), DNA repair mechanisms (ataxia telangiectasia mutated (ATM), Ataxia-telangiectasia and Rad3-related (ATR), and Breast cancer gene (BRCA1)), and mitochondrial functions, thereby maintaining redox balance and genomic stability and controlling apoptosis and proliferation [7].

Conventional cancer treatments like chemotherapy and radiotherapy induce tumor cell death by generating excessive ROS but often cause significant side effects and risk of secondary cancers [8,9]. Growing interest in alternative therapies has fueled research into plant-based compounds, valued for their lower cytotoxicity and selective anticancer activity [6]. Phytochemicals are bioactive compounds found in plants that have demonstrated many pharmacological properties including antioxidant action, organ-protective and anticancer properties [10,11]. Multiple phytochemicals have been confirmed for anticancer activity due to their increased antioxidant properties which help in scavenging free radicals. Antioxidant phytochemicals may exert their antitumor effect, by blocking cell proliferation and promoting cancer cell apoptosis, and target tumor stem cells by regulating the redox homeostasis [12]. Phytochemical-induced development of ROS causes oxidative stress (OS), leading to the induction of apoptosis and the genetic and cellular damage of cancer cells. Further, phytochemicals regulate ncRNAs and modulate key molecular pathways involved in oxidative stress and cancer progression [13]. Hence, in this review, we have discussed the involvement of the OS–ncRNA axis in cancer initiation and development along with the role of phytochemicals in modulating this crosstalk for chemoprevention.

## 2. Methodology

We searched the database for “redox homeostasis”, “oxidative stress”, “reactive oxygen species”, “reactive species”, “antioxidants”, and “pro-oxidants” separated by the operator “or”. We also added the keywords “carcinogenesis”, “oncogenesis”, “cancer”, “tumor”, “tumorigenesis”, “metastasis”, “non-coding RNA”, “ncRNA”, “long non-coding RNA”, “lncRNA”, “microRNA”, “miRNA”, “Circular RNA”, and “CircRNA” separated by the operators “OR” and “AND”. For the dual role of phytochemicals and the OS–ncRNA axis, we used all the keywords along with phytochemicals and the proper operators. We limited our search from 2000 to 2025.

## 3. Redox Homeostasis and Oxidative Stress-Induced Oncogenesis

**Figure 1:**
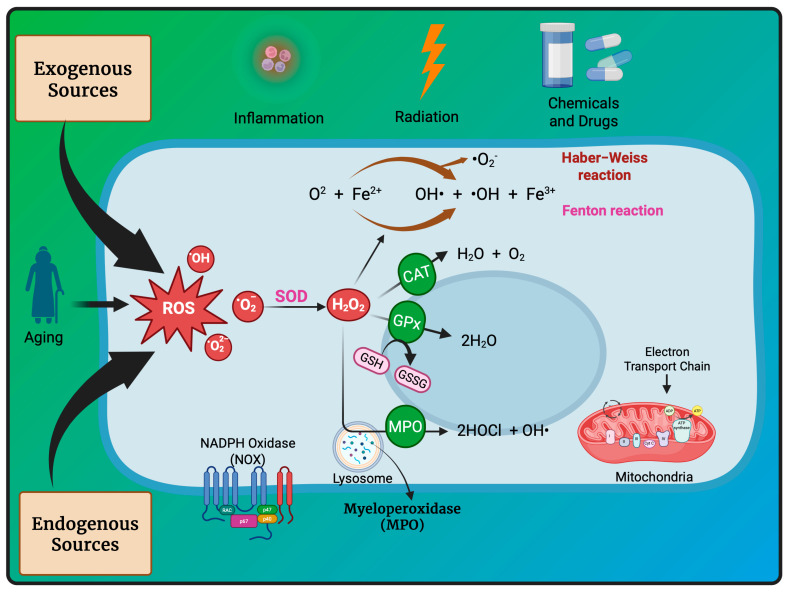
ROS sources and principal ROS molecules required in signaling. ROS generation is a series of reactions instigated by the O_2_•– production inside cells, subsidized by the exogenous and endogenous stimuli. O_2_ is reduced to O_2_•− enzymatically by nitric oxide synthases (NOS) and NOX or as redox-reaction by-products in mitochondrial reactions.

Oxidation–reduction (redox) reactions play a fundamental role in sustaining life. Reactive oxygen, nitrogen, and sulfur species regulate essential cellular processes through redox mechanisms. These reactions help control various biological functions critical for cellular survival and function [14]. Aerobic metabolism constantly produces ROS as undesirable by-products in cells. Under normal situations, the cellular antioxidant defense system effectively scavenges a low level of ROS. Some inducing factors, such as hormones, neurotransmitters, and growth factors, use ROS to carry out a normal physiological response [15]. However, any imbalance in ROS generation and cellular antioxidant mechanism results in an OS state, leading to the genesis of several diseased conditions like cancer [12]. Reactive species are effective oxidizing agents, being able to damage DNA and other biomolecules. Oxidants and free radicals are the end products of most metabolic and physiological processes. Various exogenous and endogenous factors act as a source of ROS production in the body. The body has protective antioxidant machinery; that may differ depending on the cell and tissue types and function synergistically or antagonistically (Figure 1).

Due to the harmful effects of ROS, the endogenous antioxidant system has to regulate their levels. The antioxidant machinery includes various endogenous enzymes, such as catalase (CAT), superoxide dismutase (SOD), and the glutathione system. Also, low-weight antioxidants like carotenoids, vitamins, polyphenols, etc., help regulate redox homeostasis [15].

OS is a biological phenomenon that arises due to the increased imbalance between the ROS generation and the antioxidant system. Excessive ROS production leads to substantial irreversible damage to DNA, such as double- or single-strand breaks, DNA cross-links, and base modifications that finally lead to the death of the cell [16]. Cells and tissues are also armed with detoxifying enzymes responsible for the metabolic deactivation and the successive removal of cancer-causing agents [17]. However, constantly increased levels of ROS activate other redox-sensitive transcription factors that can work as molecular regulators to transform normal typical cells into pre-malignant cells, with continuous clonal expansion into solid tumors [18]. Similarly, Acharya et al. [19] have discussed that ROS play a crucial role in gene modulation. However, while low ROS levels may be beneficial to cells, excessive ROS accumulation may alter cell signaling pathways via transcription factors.

ROS are involved in the three phases of carcinogenesis, i.e., initiation, development, and progression. ROS initiate the mitochondrial or nuclear DNA mutations leading to cancer development, including deletions, point mutations, and translocations [20]. At the initiation stage, typical normal cells are transformed into cancerous ones. After this, the activated cells are extended in the development stage to form colonies, followed by the proliferation of cells and/or apoptosis inhibition [21]. Elevated levels of OS due to lipid and DNA damage induces the tumorigenic transformation in cells, which further results in differentiation and clonal expansion due to mutations and an increased genetic instability in essential oncogenes and tumor suppressor genes, avoidance of apoptosis, increased cell proliferation, and enhanced angiogenesis [22].

**Figure 2:**
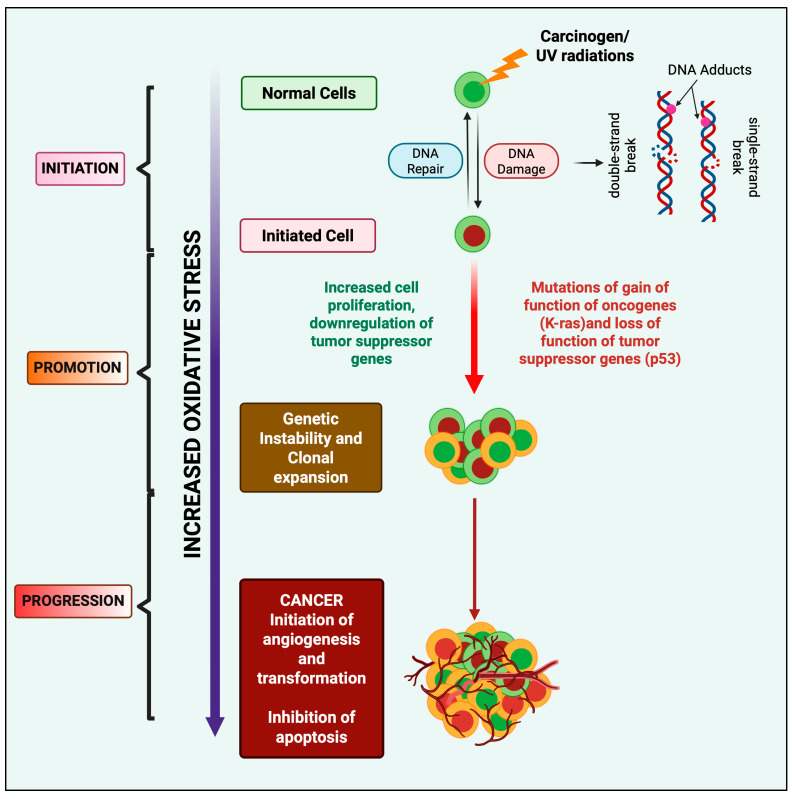
Relationship between carcinogenesis and OS: chemicals and radiation cause.

OS influences the initiation and development of cancers and tumors because they can cause damage to macromolecules like lipids and nucleic acids through the formation of free radicals, which in turn might result in genetic mutation [22], including single- or double-strand DNA breaks and eventually affecting the functions of transcription factors, signal transductors, and tumor suppressor genes such as p53 (Figure 2). The inactivation of these genes can accelerate the expressions of proto-oncogenes [22], which can result in carcinogenesis. Oxidative damage to biomolecules or genetic alterations leading to the formation of certain defective enzymes could not repair mutations, increasing the incidence of age-dependent cancers [23]. Alternatively, anticancer drug treatment and radiotherapy increase the ROS and decrease the content of antioxidants, resulting in extreme OS and apoptosis of cancer cells. However, these mechanisms of actions are equally applicable to normal cells, resulting in extreme side effects and a redundant lifestyle [22], whereas persistent OS at sub-lethal rates at critical levels lead to apoptosis resistance [24]. Furthermore, genetic changes disrupt the normal regulation of the cell cycle, apoptosis, and DNA repair processes. As a result, genomic integrity is compromised, leading to the activation of oncogenes and the inactivation of tumor suppressor genes. This creates an environment favorable for uncontrolled cell growth and the development of tumors [25].

**Figure 3:**
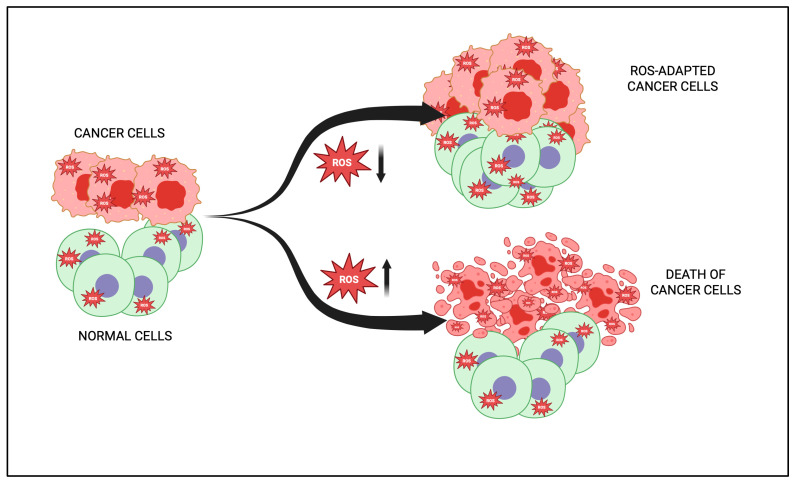
There are two promising ROS-associated anticancer therapeutic approaches. First, it involves decreasing the ROS levels to neutralize them and, thereby, using cell transformations, aiming to lower the transforming cells’ content, to starve them (upper right side). Second, based on the thought that cancerous cells, having antioxidant machinery previously triggered, were further subtle than normal cells, this approach involves increasing the ROS level and being determined to attain a redox equilibrium. Hence, by ROS induction under metabolic situations, a significant population of cells results in death (lower right side).

The human papillomavirus (HPV) promotes carcinogenesis by inducing oxidative stress (OS), which causes genomic instability and DNA damage, and facilitates viral integration into the host genome. OS disrupts mitochondrial function to elevate ROS levels and modulates antioxidant enzymes like superoxide dismutase (SOD), reduced glutathione (GSH), and glutathione peroxidase (GPx). It also drives chronic inflammation and activates key signaling pathways such as PI3K/AKT/mTOR and ERK [26]. Two possible mechanisms can involve active oxygen in the carcinogenesis process. One is the induction of gene mutations due to cellular damage, and the second is the alterations in the transcription and signal transduction factors. These mechanisms are generally related to factors such ROS and stress intensity [25]. Evidence suggest that there is an increased risk of mutation in the presence of free radicals for a number of telomeric genes, including cell cycle-related genes like p53. Additionally, various cellular intermediates influence cell signaling pathways through transcription factors, including signal transduction and transcription activator (STAT)-3, nuclear factor-kappa B (NF-κB), kinases, hypoxia-inducible factor (HIF)-1α, various cytokines, and other proteins and growth factors [27]. The primary mutation in the p53 repressive gene is the transversion from G to T in humans. Interestingly, oncogene (KRAS) and tumor suppressor gene (TP53) mutations found in smokers’ lungs that are exposed to tobacco smoke were also associated with OS and AN increased amount of 8OHdG DNA adducts [28]. Finally, increased levels of ROS lead to carcinogenesis progression by inducing genomic instability, which enhances the tumor cells’ metastatic efficiency. It has been shown that isolated cancer cells from blood or secondary cancer sites exhibit higher mitochondrial and cytoplasmic-derivative ROS rates than those from primary tumors [29]. OS causes basement membrane and extracellular matrix (ECM) disintegration by upregulating matrix metalloproteinases (MMPs) through ROS-induced proteolytic activity [30]. In addition to the damage to the ROS-mediated DNA, lipid peroxidation promotes carcinogenesis in the presence of high ROS levels. Generally, the concentration of 4-hydroxynonenal (4HNE) in the cell, a significant lipid peroxidation end-product, has been known to generate DNA adducts in TP53 in liver carcinoma [31] and promote the growth and development of the colon and kidney [32]. Cumulative numbers of beneficial approaches were being established to reduce the levels of ROS to overcome incompatibility with redox alteration in cells prompting OS (Figure 3).

According to Schumacker [33] and Fruehauf and Meyskens [34], the phrase “live by the sword, die by the sword” and “breath of life and death,” respectively, best describes the function of ROS in cancer cells. The dual function of ROS can help in fighting cancer. Pro-oxidant strategies, which contrast with traditional antioxidant therapies, offer a promising approach for anticancer drug development. Combining agents that elevate ROS with those that suppress antioxidant defenses has shown effectiveness against blood cancers with minimal impact on normal cells and has yielded encouraging results in human trials for breast, pancreatic, and lung cancers [35]. The utilization of nutraceuticals or derived compounds separate or in conjunction with chemotherapy has been used for years with promising results pre-clinically, clinically, and anecdotally. ROS-generating natural compounds like resveratrol, Lupeol, Apigenin, curcumin, and catechins are capable of efficiently killing cancerous cells [15]. Watson [36] noted that the bulk of the substances used to destroy cancer cells either directly or indirectly generate ROS that obstructs critical cell cycle phases, suggesting the role of ROS in cancer therapeutics and management. Additionally, activating cellular antioxidant systems through the upregulation of Nrf2-regulated genes, repressing the proliferation of injured or triggered cells through the downregulation of AP-1or NF-κB, or both tend to be realistic processes for attaining chemoprevention. Focus has recently been placed on intracellular signaling pathways, which control the differentiation and proliferation of cells and are thought to be a significant contributor to the development of cancer.

## 4. Dual Role of Phytochemicals in Redox Modulation

Phytochemicals consist of several biologically active compounds constituting alkaloids, polyphenols, nitrogen, carotenoids, etc. These are found in plant products and are responsible for the plant’s distinct characteristics, such as smell, color pigmentation, protection against pathogens, and other harmful agents [37,38]. Phytochemicals as a dietary resource contribute positively to health and have gained attention as alternatives to synthetic medicines for managing long-term, non-communicable diseases like cancer and diabetes. They exhibit a wide range of biological activities, including anti-inflammatory, antibacterial, anticancer, antidiabetic, antifungal, and antioxidant effects. Research linking dietary antioxidants to non-communicable disease prevention highlights the potential of plant-based foods and phytochemicals in safely reducing cancer risk and promoting overall health [39,40]. Because of their antioxidant properties, carotenoids and flavonoids are important phytochemicals undergoing intense research. Besides, they have various preventive medical benefits, such as maintaining inflammation balance, lowering the risk of certain cancers, and amplifying neurocognitive, cardiovascular, bone, and eye health in humans [41]. They exist in small amounts in plants and have pharmacological action. Phytochemicals can pose negative and positive effects on health depending on their doses [41]. The phenolic compounds can escape from digestion in the upper digestive tract and be absorbed into blood during digestion. Hence, they can provide a beneficial role in health [42]. The polysaccharides present in fruits and vegetables possess an anti-inflammatory as well as analgesic activity that can increase the level of reactive nitrogen species (RNS) and increase the levels of antioxidant enzymes (SOD, GPx, and Catalase (CAT)) [42,43,44]. Polyphenols and carotenoids are strong antioxidants that can easily neutralize free radicals and thereby decrease oxidative stress [45]. Flavonoids decrease oxidative stress by inhibiting inducible nitric oxide synthase (iNOS) and xanthine oxide synthase or can regulate the ionic channels and reduce low-density lipoprotein (LDL) oxidation. Apigenin decreases the levels of oxidative stress markers, such as glutathione peroxidase, superoxide dismutase, and activate caspase-3. Certain flavonoids display anticancer activity (cytotoxic as well as cytostatic) in cancer cells [46]. The anti-inflammatory property of flavonoids helps them in reducing the activity of inflammatory molecules [42]. Other major phytochemicals, such as glucosinolates, which are found in broccoli and kale, have anticancer properties. Glucosinolates activate and upregulate anticancer enzymes and help in the removal of carcinogens and decrease the risk of cancer [47]. Cocoa and coffee are rich in alkaloids and stimulate the central nervous system [48]. Phytochemicals via different mechanisms reduce oxidative stress, regulate the expression of genes, strengthen the defense of cells, and decrease inflammation [49,50,51,52]. Several studies have shown that phytochemical-rich diets can decrease the risk of chronic diseases, including cancer [53]. It has been shown that increasing the intake of fruits and vegetables is associated with reducing the chances of various cancers and heart diseases [54,55]. These phytochemicals have the ability to repair cell and DNA damage, thereby maintaining the genomic stability and averting cancer-causing mutations [40]. Phytochemicals with their antioxidant properties can neutralize ROS and regulate antioxidant enzymes, preventing the cells from further oxidative damage [49,56]. Phytochemicals, through mechanisms like apoptosis induction, prevention of angiogenesis, and cell proliferation inhibition, help prevent cancer [57,58]. These phytochemicals hinder cancer cells’ signaling pathways that make them important in cancer therapy and research. They upregulate the expression of pro-apoptotic molecules, such as Bax, and downregulate the expression of anti-apoptotic proteins like Bcl-2 and thereby promote cancer cell death [57].

Curcumin modulates the expression of several cancer targets, including cyclooxygenase-2 (COX-2), nuclear factor kappa B (NF-κB), STAT-3, tumor necrosis factor-α (TNF-α), and cyclin D1 and is found to decelerate cell growth through cell death and cycle arrest [38,59]. The anticancer and anti-proliferative effects of resveratrol against various tumor types are supported by multiple research studies that advocate for the impact of resveratrol in several phases of tumor initiation and proliferation through many signaling pathways and in the induction of cancer cell death [60]. Catechin polyphenols, mainly EBCG and EGCG, possess strong anti-inflammatory and antioxidant activity and impart an anticancer role by preventing DNA damage, thereby avoiding mutagenesis in normal cells [38,61,62]. Another phytochemical, lycopene, enhances the expression of endogeneous antioxidant proteins such as glutathione-S-transferase (GST)-Ω1 and SOD-1 and decreases the expression of ROS-producing proteins, thus decreasing the generation of ROS. In addition, it also possesses anti-proliferative and apoptotic activity against cancer cells [63,64]. Caffeic acid (CA) has antioxidant, anti-inflammatory, anticancer, neuroprotective, and immunomodulatory activities. It exerts an antioxidant effect by inhibiting DNA damage from free radicals [65,66]. Urosolic acid (UA) and its derivatives demonstrate numerous biological activities against diseases such as diabetic neuropathy, inflammatory diseases, and cancer. UA shows anti-proliferative, anti-angiogenesis, anti-metastatic, and apoptotic effects and acts as a ROS scavenger, initiating many pro- and anti-apoptotic proteins [67].

## 5. Bioavailability and Pharmacokinetics of Phytochemicals

The phytochemicals and their derivatives have been shown to regulate OS and inflammation. Still, the proper time and absorption for the best result is not adequately understood. The consumption of dietary phytochemicals and their bioavailability in the body to target tissues and cells aid in their biological activity. The body’s phytochemicals have low bioavailability because they handle them as xenobiotics. Their chemical structures and dosage are the main factors that affect their bioavailability. Their bulky structure and heterogeneity increase their time to reach maximal plasma concentration (T_max_) in the body. For instance, the plasma peak and removal time of green tea, a flavan-3-ols, is 1–2 h post-ingestion and the next few hours post-peak level, while lycopene from tomato reaches its maximum peak levels between 15 and 33 h after ingestion and clears entirely from the body within next few days [68,69]. The T_max_ of ellagic acid from pomegranate is 0.5–1 h post-ingestion in liquid form and 2–3 h in solid form. It was observed that the intake of dietary phytochemicals with different food types affects their absorption [70]; for example, increased lycopene absorption has been observed when food was taken with olive oil [71], and absorption of dietary anthocyanins and phenolic acids is compromised when it is taken with nutritional fibers [72].

The pharmacokinetic study of resveratrol provided evidence that it shows poor bioavailability because of rapid liver metabolism, thereby reducing its therapeutic effects. Many approaches have been identified to overcome this, including complexing it with piperine, preparing its nanoparticles, and using other drug delivery systems to stabilize its bioavailability [73]. The caffeic acid ingestion can reach the maximal level in plasma within 1 h. However, its level is reduced quickly, requiring repetition of its dose every 2 h. Its chemical modification can be utilized to increase its improved bioavailability, pharmacokinetic functions, and safety [74]. EGCG from green tea reaches its highest plasma concentration of 0.15 μmol/L after ingesting two cups of green tea, producing its biological effects [75]. This property can improve the effectiveness of drug delivery to the target sites [73]. The pharmacokinetic experimentations of urosolic acid at an oral dosage of 300 mg/kg have reported low plasma concentration and removal in a comparatively short time, i.e., <1 h. The pharmacokinetic profile of urosolic acid has shown that it can reach multiple tissues, such as testes, lung, colon, spleen, kidney, liver, brain, bladder, and heart [67].

Lupeol, extracted and assessed through UPLC–APCI+–MS/MS, demonstrated pharmacokinetic characteristics, including a time-to-peak concentration of 6.444  ±  0.851  h and a peak concentration of 8.071  ±  2.930  μg/mL. Investigations into direct digestion and absorption across various organs unveiled notable Lupeol concentrations in the stomach to be 137.25  ±  19.94  ng/mg and in the small intestine to be 99.00  ±  12.99  ng/mg during the early post-administration period. The predominant excretion pathway was through feces, reaching its peak at 12 h post-administration (163.28  ±  9.83  μg/mg). Despite its hydrophobic nature, Lupeol demonstrated absorption in animals exceeding expectations, reflected in the extent of absorption (F) of 0.645  ±  0.0581 [76]. The pharmacokinetic profiles of berberine were investigated in rats following both intravenous (4.0 mg/kg) and oral administration (48.2, 120, or 240 mg/kg). Berberine demonstrated a low absolute bioavailability of 0.37 ± 0.11%. It underwent rapid metabolism, detecting all nine metabolites in vivo. Excretion studies, employing a single intragastric dose of 48.2 mg/kg Berberine, revealed elevated AUC0–48 h values for phase II metabolites compared to phase I metabolites, suggesting the predominant presence of phase II metabolites in circulation. Fecal excretion constituted 18.6% of berberine, primarily as berberrubine (M1). The total recovery of berberine and its metabolites from urine, bile, and feces was 41.2% [77].

Curcumin’s therapeutic effectiveness is hindered by its poor bioavailability after oral intake. This is primarily due to its low solubility in water (around 11 ng/mL), limited absorption in the gut, rapid metabolism, and fast excretion. In the intestines, liver, and kidneys, phase 1 metabolism transforms curcumin into various metabolites like dihydro-, tetrahydro-, hexahydro-, and octahydro-curcumin. These metabolites undergo phase 2 conjugation reactions, forming highly soluble glucuronide and sulfate conjugates eliminated via feces and urine. Gut microbiota also participate in curcumin metabolism through microbial reductase pathways. Despite high oral doses, curcumin plasma concentrations remain low, typically 21–41 ng/mL in cases such as advanced pancreatic cancer, where patients receive 8 g daily. Notably, the predominant forms found in plasma are glucuronide and sulfate conjugates, which do not possess the same bioactivity as free curcumin [78].

## 6. Pharmacological Effects of Phytochemicals

### 6.1. Anti-Inflammatory Effects

Inflammation has an important role to play in multiple diseases like diabetes, cancer, neuropathy, etc. Organ injury induces an inflammatory reaction to recuperate homeostatic equilibrium; however, relentless inflammation can increase the ROS production in the respective tissue which in turn upregulates tumor-promoting genes [11]. Inflammation and OS facilitate cancer development in multi-phase which includes direct injury to nuclear DNA and modification of intracellular signaling pathways involved in inflammation such as protein kinase C (PKC), protein kinase b (PKB), tyrosine kinases, phosphoinositide 3-kinase (PI3K), MAPKs, glycogen synthase kinase, etc. These signaling pathways also trigger redox-related transcription factors such as Nrf2, NF-κB, or AP-1 [79].

Phytochemicals can be used as a natural modulator of pro-inflammatory gene expressions. The phenolics and triterpenoids in fruits and vegetables possess strong anti-inflammatory properties. Lectins and peptides typically displayed anti-inflammatory action in dietary beans [43]. Several studies have shown the anti-inflammatory properties of phytochemicals. These phytochemicals modulate critical inflammatory signaling pathways, including NF-κB, STAT, MAPKs, and Nrf-2 [80]. Caffeic acid displays anti-inflammatory properties by inhibiting NO generation and blocking the production of iNOS and COX-2 [81]. EGCG shows its anti-inflammatory action by blocking the activation of AP-1, NF-κB, Toll interleukin-1 receptor, and the MyD88-dependent pathway and blocking the expressions of NO synthase, TNF-α, and COX [82,83]. NF-κB is activated due to ROS, thus supporting the biosynthesis of COX, NO, and TNF-α. The generated ROS can be scavenged by EGCG, causing anticancer effects [74]. Ursolic acid and its analogs possess anti-inflammatory potential, block the action of inflammatory cytokines such as COX and iNOS expression and antioxidant properties like Nrf2 signaling activation [11,84]. Curcumin nanoparticles exhibit anti-inflammatory properties by downregulating the serum levels of IL-1β, IL-6, TNF-α, LFT, RFT markers, and lipid profiles while increasing high-density lipoprotein and IL-10 levels [85]

### 6.2. Antioxidant Effect

In addition to being crucial in preventing and treating chronic diseases, phytochemicals also play a significant role in boosting overall body health by acting as free radical scavengers, regulating metabolism, postponing senescence, and other important bodily functions. Due to their advantageous benefits and antioxidant activity, phytochemicals have steadily attracted prominence in nutritional research [86]. Naturally occurring antioxidants known as phytochemicals are among the most promising components employed in diets today. The antioxidant response element/nuclear factor (erythroid-derived 2)-like-2 transcription system provides a molecular basis for the antioxidant activities to clarify their underlying mechanisms that target signal transduction pathways [87]. CA has shown α-tocopherol inhibition in low-density lipoprotein (LDL). It inhibits lipid oxidation and possesses photo-protective properties against UV radiation [66]. EGCG possesses substantial antioxidant properties and is involved in the modulation of GSH metabolism, ROS generation, and cytochrome P450 2E1 action. It blocks tissue injury by inhibiting the production of pro-oxidant enzymes, like xanthin oxidase and COX, and reduces the action of MDA, IL-1β, protein carbonylation and MPO while increasing the expression of endogenous antioxidant enzymes, such as antioxidant heme oxygenase-1, GSH, and SOD [74,88]. Laboratory analysis showed that EGCG shows action in two phases and functions as a pro-oxidant depending on the cellular environment and its concentration [89]. However, it is reported that, at lower concentrations, it shields cells from the adverse effects of OS [90].

## 7. Anticancer Action of Phytochemicals

The specific processes by which phytochemicals execute anticancer activity are yet a point of research. These can straightforwardly retain the ROS or advance the concentration of antioxidants (e.g., CAT, SOD, and glutathione) in a cancerous cell. A phytochemical can smother harmful changes in a pre-tumorous cell or hinder the cancer-causing agent’s metabolic transformation. Table 1 lists some prominent phytochemicals and their mode of action against cancer.

Additionally, they can regulate cell and signaling pathways engaged with malignant cells’ development, intrusion, and metastasis. Ellagic acid extracted from pomegranate induces prostate cancer cell apoptosis via activation of caspase-3 and restrains metastasis in ovarian cancer via MMP2 and MMP9 downregulation [140,141]. EGCG stifles the de novo lipogenesis pathway that drives cancer cell proliferation in an aggressive nature. Interestingly, EGCG drives cancer cell death and cytotoxicity while sparing normal cells [142]. Flavanones, lignans, and isoflavones forestall estrogen signaling to cancer cells and decrease their multiplication [143]. Besides these systems, the anticancer phytomolecule focuses on a few different signaling pathways to diminish the development and metastasis of cancer cells.

Curcumin acts as an antioxidant and increases an enzyme’s activities [6]. Curcumin incubation increases cell resistance towards damage through OS [144]. Previous research revealed that curcumin avoids lipid peroxidation and breakages of the DNA strand [145]. It was reported that curcumin induces antioxidant enzymes like glutathione peroxidase, glutathione reductase, glucose-6-phosphate dehydrogenase, and CAT in phase II and augments the natural antioxidant machinery of the body, leading to mutagens and carcinogens’ detoxification [146]. It also lowers the formation of nitric oxide, the primary component in inflammation and carcinogenesis [147]. Resveratrol is a renowned antioxidant nutraceutical. Curcumin inhibits breast cancer’s progression, proliferation, and invasiveness by targeting the EGFR signaling pathway. Curcumin reduces the level of epidermal growth factor receptor (EGFR), Akt, and matrix metalloproteinase (MMP2) while upregulating tissue inhibitors of metalloproteinase (TIMP-1) in breast cancer. Further, curcumin modulates oncogenic and tumor-suppressive miRNA to induce apoptosis in breast cancer [148]. In prostate cancer, curcumin represses the activation of NF-κB by suppressing the mitogen-activated protein kinase (MAPK) and EGFR pathway, which in turn downregulates pro-apoptotic genes such as Bcl-2 and Bcl-xL, IL-6, cyclooxygenase (COX)-2, and C-X-C motif chemokine ligand (CXCL) 1 and 2 [149]. Another study observed that a curcumin analog could inhibit gastric cancer by targeting glycolysis through the ROS–YAP–JNK signaling axis. It was observed that curcumin analog-induced inhibition of glycolysis and overproduction of ROS results in the activation of Janus kinase (JNK) and repression of yes-associated protein (YAP), which further leads to the death of cancer cells [150]. Curcumin administration can increase OS in the tumor microenvironment. Curcumin can tether with many enzymes, such as carbonyl reductase, nicotinamide adenine dinucleotide phosphate (NADP), and glutathione s-transferase (GST)-P1, resulting in increased mitochondrial damage, ROS generation, and cancer cell death. The results can be enhanced by combining curcumin with drugs, delivery systems, nanocarriers, or derivatives [151].

Various other plants are being profiled for their activity against cancer cells [152]. Pintea et al. [153] studied resveratrol’s protective role against H_2_O_2_-mediated OS in human RPE cultured cells. They discovered that resveratrol does not have a cytotoxic effect in culture media at 25–100 μM concentrations but displayed a prophylactic effect against cytotoxicity induced by H_2_O_2_. Resveratrol pre-treatment induced significant, dose-dependent increases in the activities of SOD, GPx, and CAT. It also increased the reduced glutathione level, both under basal and OS. Spanier et al. [154] has reported that resveratrol’s prophylactic effects against OS are due to the upregulation of intrinsic antioxidant mechanisms rather than ROS direct scavenging activities. Resveratrol exerts its anticancer activity by modulating tumor growth factor (TGF)-β/ Suppressor of Mothers against decapentaplegic (SMAD). In colorectal cancer, administration of TGF-β can lead to decreased EMT as observed by downregulated E-cadherin and upregulated Vimentin expression, which further results in reduced lung and hepatic metastases [155]. In another study, it was reported that resveratrol can inhibit cell viability and proliferation in lung cancer cells, resulting in an apparent increase in apoptosis.

Further, resveratrol inhibits lung cancer by inducing G1 phase arrest and modulating apoptotic proteins such as Bcl-2, Bax, and cleaved caspase-3. Interestingly, increased exposure time to resveratrol results in the accumulation of ROS and activation of downstream antioxidant signaling pathways [156]. Also, in pancreatic cancer, resveratrol inhibits HIF-1α and associated abnormalities [157]. Resveratrol can potentially combat lung cancer by modulating multiple molecular targets and signaling pathways. These include pathways related to OS, inflammation, apoptosis, and autophagy, as well as influencing the expression of microRNAs [158]. Farhan et al. [159] reported that resveratrol extracted from red grape and pomegranate can reduce cell viability and induce cell death in prostate cancer by mobilizing nuclear copper and ROS generation, causing DNA damage. As observed by the author, prostate cancer cell death by resveratrol can be reversed by administering a copper chelator, neocuproine, and a ROS scavenger.

EGCG from green tea exhibits many biological and pharmacological activities, including antioxidant actions, iron-chelation capacities, and lipid peroxidation attenuation by various radicals. It is believed to be an antioxidant in cellular systems [160]. EGCG exhibits anticancer activity via the reduced expression of a Toll-like receptor 4 and upregulation of a Toll-interacting protein via a 67-kDa laminin receptor (67LR). Further, administration of EGCG results in a reduced NF-kB DNA binding property and repression of interleukin (IL)-1b, IL-6/8, and tumor necrosis factor (TNF)-a. Also, EGCG induces apoptosis in breast cancer cells ([161], . Similarly, in multiple myeloma cells, ECCG induces apoptosis through upregulating 67LR. Further, lipid-raft-dependent apoptosis has also been observed where activation of acid sphingomyelinase results in the degradation of sphingomyelin and lipid raft clustering [162]. In another study, it was observed that EGCG can inhibit Stat3 and nuclear factor-kappa B (NF-kB) in cancer cells, resulting in the decreased expression of VEGF in breast cancer cells. The repression of signal transducer and activator of transcription (STAT)3/NF-kB/vascular endothelial growth factor (VEGF) leads to decreased angiogenesis, reduced tumor weight, and cancer cell proliferation [163]. In an investigation, EGCG  +  DATS exhibited a concurring effect by downregulating cellular migration and facilitating intracellular ROS levels. This combination also promoted cell apoptosis characterized by phosphatidylserine externalization, condensed cell morphology, and increased cell cycle arrest [164].

Apigenin plays an essential role in the chemoprevention of cancer as well as in chemotherapy. It has been demonstrated to stop lipid peroxidation and protect the antioxidant machinery in animals administered with N-nitroso diethylamine [165]. Apigenin has been found in culture and tumor models to neutralize free radicals and stimulate detoxification enzymes (phase II) [166]. Apigenin decreases intracellular ROS and induces apoptosis and autophagy in primary effusion lymphoma. Further, Apigenin inhibits the pro-survival pathway by activating p53, which further increases CAT activity and inhibits STAT3 [167]. In addition to that, Apigenin induces cell death and autophagy in HCC cells by suppressing the phosphoinositide 3 kinase (PI3K)/AKT/mammalian target of Rapamycin (mTOR) pathway. Also, Apigenin treatment in prostate cancer results in lower levels of phosphorylated FoxO3a and AKT, which results in reduced nuclear retention and binding [165]. Shi et al. [168] have reported that Apigenin acts as a pyruvate kinase M2 inhibitor, leading to increased apoptosis and autophagy in colorectal cancer, suggesting its role in cancer chemoprevention. Research has demonstrated that Apigenin influences cancer stem cell (CSC) metabolism and diminishes CSC populations through several mechanisms. These include suppressing the Wnt/β-catenin signaling pathway, inhibiting NF-kB protein expression, and reducing cell cycle activity by upregulating p21 and cyclin-dependent kinases [167,169]. Apigenin inhibits cell growth in both a dosage and time-dependent manner, leading to reduced clone formation and the promotion of apoptosis. These effects are mediated through the Akt/Bad/Bcl2/Bax axis, which triggers the mitochondrial pathway of apoptosis, thereby limiting cell proliferation.

Moreover, Apigenin’s anti-proliferative effects on gastric cancer cells appear particularly potent in aggressive phenotypes. However, it also affects normal gastric cells. Consequently, careful dosage considerations are crucial to mitigate potential side effects on the normal gastric epithelium. Further, Apigenin inhibits ROS production, downregulating NF-kB and mucosal damage of the gastric lining. These damages can lead to gastric cancer [170]. Interestingly, Apigenin can also induce OS in cancer cells to trigger apoptosis. In various cancers, Apigenin facilitates ROS accumulation, lowers antioxidant defense system, and decreases mitochondrial integrity to cause cancer cell death. Studies have shown that Apigenin administration in HCC cells causes downregulation of CAT and GSH [170].

Lupeol is an important triterpenoid found in many plants and fruits and has anticancerous effects. It has been observed that Lupeol exerts an anti-metastatic effect through the repression of the MAPK/ERK signaling cascade in lung cancer. Further, a significant decrease in the expression of N-cadherin and Vimentin at the genome level has been observed [171]. In another study, Eldohaji et al. [172] reported the anticancer effect of Lupeol in HCC cells. They observed that Lupeol represses the expression of the anti-apoptotic protein *BCL-2* and promotes the cleavage of caspase 3. Further, Lupeol promotes G1 phase arrest, thereby actively suppressing the proliferation of liver cancer cells. Lupeol induces chromatin condensation, PARP cleavage, and G1 cell cycle arrest in non-small lung carcinoma. Further, Lupeol modulates the activity of STAT3 and reduces the activation of associated downstream genes [173]. In a detailed meta-analysis by Fatma et al. [174], the authors concluded that Lupeol effectively reduces tumor volume and weight. Moreover, their findings suggest that combining Lupeol with other chemotherapy agents, such as Dacarbazine, Cisplatin, Doxorubicin, etc., holds the potential for augmenting anticancer effects. Lupeol was also found to target various molecular pathways, resulting in cell cycle arrest, apoptosis, inhibition of angiogenesis, reduced inflammation, and maintenance of redox homeostasis. In NSCLC, Lupeol and a few other pentacyclic triterpenes showed cytotoxicity via inducing cell cycle arrest, ROS, apoptosis, and mitochondrial depolarization. Further downregulation of STAT3 and programmed death-ligand 1 (PDL1) genes was also observed [175].

**Figure 4:**
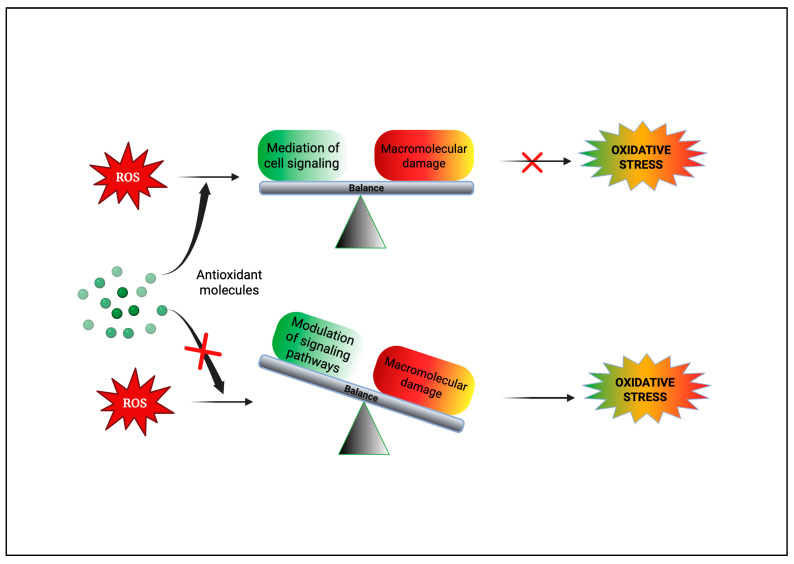
Figure portraying the balance between ROS production and antioxidant defense.

Epidemiological research has reported that fruit and vegetable consumption regularly decrease the risk of developing chronic conditions like cancer [176]. In a longitudinal study, Serafini [177] reported that elevated intakes of antioxidant-rich root vegetables, fresh fruits, and vegetables were correlated with a decline in death rate and nutraceuticals that were abundant in antioxidants have a protective impact on cancer (Figure 4). Although the biological actions and metabolism of polyphenols in the body are not fully known, there is an agreement that flavonoid’s free radical scavenging activity seems to be a mixture of chelating metals and antioxidant properties [177].

The polyphenols’ structure allows them to have antioxidant activities. The extent of methoxylation and hydroxyl groups were significant factors that allowed them to show antioxidant activities. Recently, it has been reported that the naringenin-oxime compound has additional anti-genotoxic and antioxidant efficiency compared to naringenin present in human mononuclear leukocyte cells with a few hydroxyl groups [178]. The oxidases, like myeloperoxidase (MPO), lipoxygenase (LO), xanthine oxidase (XO), and NADPH oxidase, were known as the significant mechanisms through which these phytochemicals inhibit the generation of large levels of ROS [179]. By enhancing well-known antioxidant enzymes like CAT and SOD, these phytochemicals can also disrupt enzymes that were indirectly implicated in the oxidative cycle [180]. Due to this, flavonoids may be regarded as phytochemicals that may indirectly or directly interfere with the generation of ROS [181].

One principal feature of cancerous cells is their survival ability. Hence, cancer therapy’s primary aim involves destroying cancerous cells without destroying normal cells. Chemotherapy, radiation, and surgery are a few of the treatment options for cancer. For cancer patients, chemotherapy is the primary form of treatment [182]. While it is intended to kill the targeted primary cancer cells, normal cells are also impacted, resulting in multiple side effects in different organ systems [183]. For this purpose, attempts were made to find new and efficient forms of treatment. The research is focused on finding the active compounds of low-toxic plants and their high selectivity to destroy cancerous cells. Most drugs targeted against cancerous cells have the primary mechanism of creating ROS [184]. There has been a practical approach for treatments aiming dramatically at elevating intracellular ROS by reducing the antioxidant ability to kill cancer cells [185]. This could be accomplished by utilizing compounds that suppress antioxidant machinery or by suppressing different signaling pathways, which can improve antioxidant control in cancerous cells. The high level of ROS can induce cancer cell apoptosis through deleterious ROS functions or by activating apoptosis-specific death-signaling pathways. The chemotherapeutics that generate ROS include anthracyclines (epirubicin and doxorubicin), alkylating agents (cyclophosphamide and melfalan), derivatives of podophyllin (etoposide), camptothecin (irinotecan and topokan), and complexes of platinum coordination compounds (carboplatin and cisplatin) [186]. Elevated ROS levels lead to acute damage to cell components, including proteins, lipids, and DNA. Because of its high reactivity, ROS may damage DNA. It can oxidize DNA bases, DNA helix damage, and DNA lesions [187]. The first drug to be produced based on features of ROS development was procarbazine. It gets hydrolyzed in water, and the drug’s cytotoxic reactions derive from the formation of H_2_O_2_ [188]. As with chemotherapy, radiotherapy also destroys cancer cells through the development of ROS [189]. Three pathways can kill cancerous cells: apoptosis, necrosis, and autophagy [190]. Apoptosis, a regulated death of cell death, may be triggered by extrinsic as well as intrinsic (in mitochondria) death receptors, and ROS are connected to both pathways [191].

**Figure 5:**
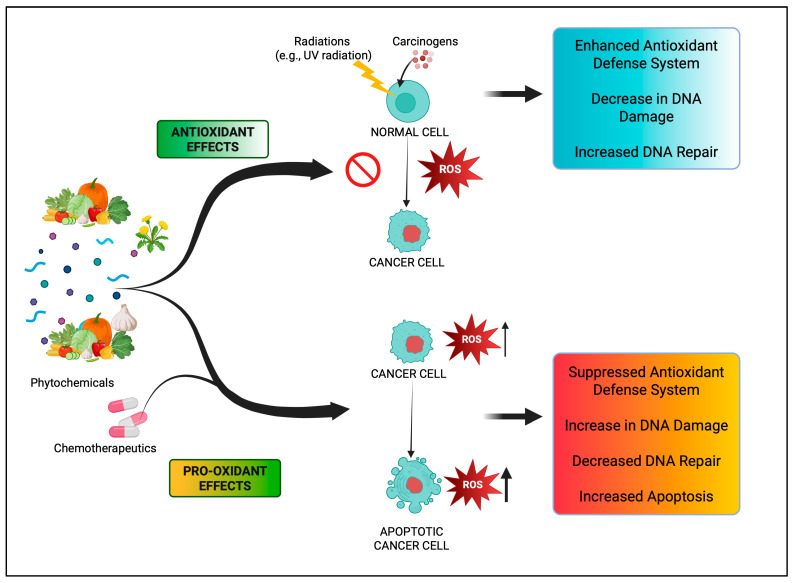
The pro-oxidant and antioxidant roles of plant compounds: Their prophylactic role occurs due to their capability to decrease carcinogens and radiation-induced ROS generation. Plant compounds activate the intrinsic antioxidant machinery, mitigate OS, and prevent macromolecular damage. When given together with chemotherapeutic drugs, they can elevate OS in cancerous cells, thereby suppressing survival signals, upregulating apoptotic signals, causing damage to DNA, and suppressing functioning pathways that drive the growth of cancer cells.

Some studies have demonstrated that ROS can function as a signaling moiety in inducing the autophagic death of cells in cancerous cells [192]. It is also essential for cancer cell survival and their capacity to kill. Besides that, the dosage, duration, forms, and position of ROS development have been linked to the capability of cancerous cells to discern ROS as the apoptotic or survival signal. In short, while the cancer cells need moderate levels of ROS to survive, extreme rates kill them [193]. While plant-based phytochemicals were known to show protective effects against cancer [194], a few studies have shown that these agents can kill cancerous cells [27]. Many medications utilized in chemotherapy today include plant products like vincristine, paclitaxel, vinblastine, mitomycin, bleomycin, doxorubicin, aclarubicin, actinomycin D, and idarubicin. Many compounds like EGCG, curcumin, genistein, camptothecin, resveratrol, perillyl alcohol, phenylethylisothiocyanate, lycopene, sulforaphane, eicosapentaenoic acid, aplidine, linoleic acid, vitamin C, and ursodeoxycholic acid are being tested clinically for the treatment of cancer ([195]; Figure 5).

It is important to note that curcumin, quercetin, and ellagic acid have been shown to stimulate ROS generation and synergistically damage DNA and cancer cell line apoptosis [196]. The effect of a combination of curcumin and imatinib (a chemotherapeutic drug) has recently been studied in one patient with cancer. It has been shown to improve effectiveness in cancer treatment [197]. Besides that, pharmacological curcumin and vitamin C dosages have been accounted for in various cancer treatments, and positive findings have been reported [198]. Studies have shown that many phytochemicals across multiple cancer cells display more cytotoxic effects than normal cells [199]. Few studies have reported that polyphenols like genistein and EGCG can kill cancerous cells with apoptosis compared to normal cells at the same dose [199]. It has also been found that a plant medicine called Ankaferd, extracted from various plant extracts, destroys cancer cells at the same doses more than average cells [200]. Yet how this differential effect happens is not clear. It may occur due to variations in cancer and normal cell metabolism. Since the cancer cell’s metabolism is more than that of normal cells, the production levels of endogenous ROS were much higher than normal cells.

Pro-oxidant phytochemical usage is emerging as a promising technique for selectively targeting tumor cells, leading to the rise in cancer cells’ ROS rates. Indeed, ROS development in cancerous cells treated with phytochemicals was significantly elevated compared to the same treatment in normal cells, and there was a positive association between cell death and ROS levels [200]. The benefit of such a strategy is that the basal ROS rates of normal cells were lower than those of cancerous cells. Thus, they were less reliant on antioxidants, which did not significantly affect them. While several experiments on the usage of phytochemicals in cancer treatment have been carried out in in vitro cell culture, the number of experimental animal models and in vivo clinical trials is limited. In in vivo studies, one major problem concerning phytochemicals was their bioavailability, absorption, and digestion. Bioavailability in many phytochemicals is very poor because of their low rate of absorption [201]. Enteral administration is also favored for treating cancer [197].

### 7.1. Non-Coding RNA–Oxidative Stress–Carcinogenesis Relationship

**Figure 6:**
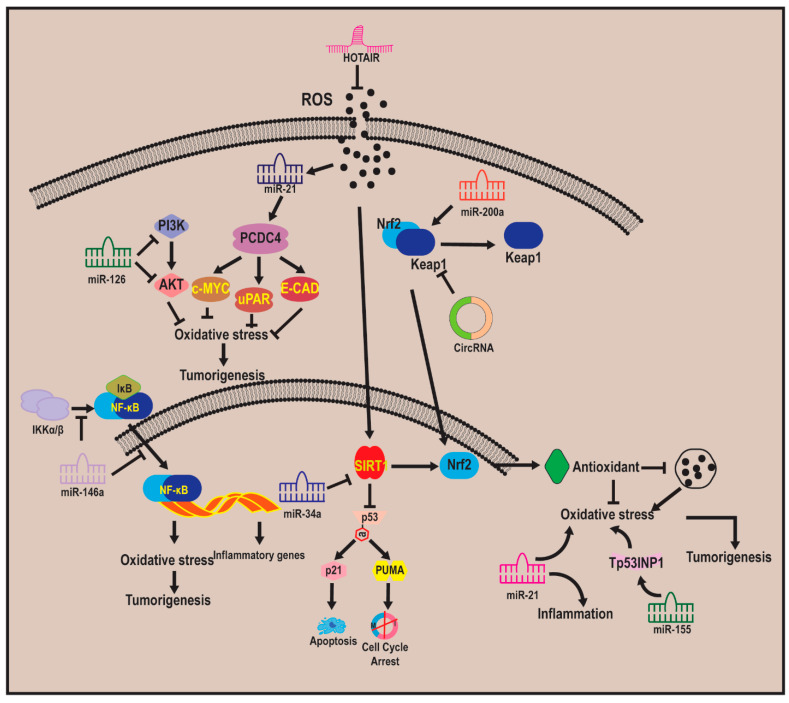
Various ncRNA regulate the level of ROS and oxidative stress which can lead to tumorigenesis.

While protein-coding genes have been extensively studied in oxidative stress responses, the role of ncRNAs has gained prominence in recent years. ncRNAs, once considered transcriptional noise, are now recognized as key regulators of cellular homeostasis. Their involvement in redox biology and cancer progression highlights their potential as diagnostic and therapeutic targets. Non-coding RNAs (ncRNAs) have emerged as critical regulators of gene expression, playing a pivotal role in oxidative stress and cancer. There is an intricate relationship between ncRNAs and their role in modulating oxidative stress in carcinogenesis (Figure 6; [202]).

NcRNAs mediate oxidative stress regulation in cancer by modulating several key cellular processes. They influence antioxidant defense mechanisms by regulating Nrf2/KEAP1 and SIRT1 signaling pathways, ensuring redox homeostasis [7]. Additionally, ncRNAs impact DNA damage response, with miRNAs and lncRNAs modulating genes involved in repair mechanisms such as ATM, ATR, and BRCA1, which are crucial for maintaining genomic stability under oxidative stress conditions [203]. Furthermore, ncRNAs exert control over mitochondrial function by regulating mitochondrial biogenesis, energy metabolism, and ROS production, which in turn affects cellular apoptosis and proliferation [204]. NcRNAs also play a role in inflammatory signaling, particularly through NF-κB and STAT3 pathways, linking oxidative stress to chronic inflammation and tumor progression [205].

By acting as molecular sponges, guides, and scaffolds, ncRNAs orchestrate the intricate balance between oxidative stress, cellular survival, and cancer development, making them attractive targets for novel therapeutic interventions. There are several classes of non-coding RNAs (ncRNAs) that have their roles in oxidative stress and cancer. These classes and their role in the oxidative stress–carcinogenesis relationship are discussed below.

### 7.2. MicroRNA

MicroRNAs (miRNAs) are short, 20–24 nucleotide-long RNA molecules that modulate gene expression post-transcriptionally. Several miRNAs have been identified as crucial regulators of oxidative stress responses in cancer [206]. miR-34a, a p53-regulated tumor suppressor, suppresses oxidative stress-induced tumorigenesis by targeting SIRT1, a key regulator of antioxidant defense [207]. On the other hand, miR-200c modulates oxidative stress by regulating KEAP1/Nrf2 signaling, thereby influencing ROS detoxification [208] . Similarly, miR-21 acts as an oncomiR by promoting oxidative stress and inflammation, leading to tumor progression [208]. The fundamental processes underpinning the ncRNAs–ROS axis-driven beginning of cancer have been uncovered by an increasing number of research. 

According to reports, hexavalent chromium and arsenic, for instance, increase ROS-dependent miR-21 transcription and impair the expression of programmed cell death protein 4 (PDCD4). This leads to the upregulation of downstream genes such as E-cad, c-myc, and uPAR, which in turn causes bronchial epithelial cells to transform malignantly [209,210]. Another miRNA, miR-155, enhances oxidative stress and promotes inflammation-associated tumorigenesis by targeting FOXO3a and TP53INP1 [211,212]. miR-126 protects against oxidative damage by regulating VEGF and PI3K/Akt signaling, affecting angiogenesis and tumor growth [213]; miR-146a also modulates oxidative stress-induced inflammation and NF-κB signaling [214], linking it to cancer progression. These miRNAs influence oxidative stress responses by targeting key regulators of redox homeostasis, apoptosis, and cellular metabolism. Dysregulation of miRNAs can exacerbate oxidative damage, leading to increased genomic instability and enhanced tumor aggressiveness. Their role as tumor suppressors or oncogenes (oncomiRs) depends on the cellular context and target genes involved.

### 7.3. Long Non-Coding RNAs

Long non-coding RNAs (LncRNAs) have an important role in the regulation of oxidative stress in various carcinomas. The level of ROS should be constantly modulated to maintain the proliferation of cancer cells, resisting chemotherapy and preventing apoptosis. LncRNAs are non-coding RNA comprising transcripts of 200 or more nucleotides which was understudied before and recently was discovered. Recent discoveries estimate that LncRNAs exceed the mRNAs by up to 20 times [215], and the variety of functions that lncRNAs undertake is also significant [216]. LncRNAs’ primary function is to regulate gene expression, enlisting multiple mechanisms: (1) lncRNAs activate gene and transcription into mRNA by acting as a bridge between transcription factors and gene promoters. (2) Gene expression is inhibited by LncRNAs by binding to chromatin regions and blocking RNA polymerase access and downregulating gene expression. (3) lncRNAs regulate genes by recruiting enzymes such as histone methylation enzymes or histone acetyl transferase, modifying the chromatin by inhibiting or promoting epigenetic changes. (4) lncRNAs act as a backbone to stabilize multi-subunit complexes that govern gene expression [217]. Additionally, lncRNAs in the cytoplasm can also affect the protein function in a post-transcriptional manner, by direct interaction with the protein (e.g., allosterically modulating enzyme activities) or by acting as competitive endogenous RNAs (ceRNAs), binding miRNAs to regulate mRNA translation [218,219]. Recent studies showed that several lncRNAs, such as HOX transcript antisense intergenic RNA (HOTAIR), metastasis-associated lung adenocarcinoma transcript 1 (MALAT1), nuclear receptor-like (NRAL), and Taurine upregulated gene 1 (TUG1), play a crucial role in the modulations of cancer cells’ oxidative stress, emphasizing a possible intersection between lncRNAs and ROS [220]. MALAT1 modulates ROS levels and redox-sensitive signaling pathways, contributing to tumor growth and resistance to therapy [220]. NEAT1 acts as a redox-sensitive ncRNA that influences p53 signaling and cellular antioxidant responses [221]. Similarly, HOTAIR, a long non-coding RNA, promotes tumorigenesis and metastasis in various tumors by regulating HOXD gene clusters. HOTAIR functions as an antioxidant regulator lncRNA and prevents the cancer cell from exceeding the ROS level that would trigger apoptosis [222,223]. Various lncRNAs modulate the ROS homeostasis at the mitochondrial level, affecting the level of ROS generated in the electron transport chain [222]. Further, by upregulating Nrf2 gene expression by acting as ceRNA for Nrf2 miRNA, Nrf2 protein degradation and nuclear inactivation can be prevented, inducing the Nrf2 antioxidant response by acting as effectors [220,224]. LncRNAs also encourage the Warburg effect in cancer cells by upregulating PKM2 isoform expression, which is another effective method that reinforces the disrupted oxidative balance [225]. Furthermore, lncRNAs cooperate with ROS in promoting cellular proliferation or apoptosis by regulating the gene expression in ROS signaling pathways (eg., TP53 and HIF-1-a) [226].

### 7.4. Circular RNAs

Circular RNAs (circRNAs) are a unique class of endogenous non-coding RNAs characterized by a covalently closed-loop structure. Unlike linear RNAs, circRNAs lack 5′ and 3′ ends, making them highly stable and resistant to exonuclease degradation. Emerging studies indicate that circRNAs play crucial regulatory roles in gene expression, acting as microRNA (miRNA) sponges, protein scaffolds, and regulators of transcription [227]. They are gaining importance because of their potential role in the modulation of tumorigenesis. They, like circFOXO3, are reported to suppress tumor growth by interacting with stress-responsive proteins [228]. CircRNA-SHPRH also regulates oxidative stress pathways and exerts tumor-suppressive functions. CircHIPK3 modulates glutathione metabolism and ROS homeostasis in cancer cells [229].

Given their critical role in oxidative stress and cancer, these ncRNAs hold great promise as therapeutic targets. The development of ncRNA-based biomarkers offers a non-invasive approach for early cancer detection, prognosis, and monitoring of oxidative stress-related malignancies. Additionally, ncRNA modulators, including small molecule inhibitors, antisense oligonucleotides, and RNA-based therapeutics, are being investigated to restore or suppress ncRNA functions to counteract oxidative stress-driven tumor progression [230]. Moreover, the combination of ncRNA-targeted therapies with conventional treatments, such as chemotherapy, radiotherapy, and immunotherapy, may enhance therapeutic efficacy, minimize drug resistance, and improve patient outcomes [231]. However, challenges such as efficient delivery systems, off-target effects, and stability of ncRNA-based drugs must be addressed to facilitate their clinical translation. Future research should focus on optimizing ncRNA therapeutics and conducting large-scale clinical trials to validate their safety and efficacy in cancer treatment.

### 7.5. Phytochmeicals That Target ncRNA–Oxidative Stress–Carcinogenesis Relationships

Various common phytochemicals are known to potentially target ncRNAs in cancer cells. Phytochemicals such as curcumin, resveratrol, sulforaphane, berberine, etc., are reported to target lncRNA [232]. Curcumin, extracted from turmeric, can modulate lncRNAs associated with human diseases. Curcumin can suppress the expression of oncogenic lncRNAs such as H19 and ROR in various types of cancer cells, simultaneously inhibiting the Wnt/β-catenin pathway [110,233,234]. Curcumin inhibits the growth of colorectal cancer cells appreciably when given to mice with silenced lncRNA such as PANDAR; the mechanism however remains underexplored. It can be inferred that knockdown of PANDAR can switch curcumin-induced senescence to apoptosis, which could be significant in colorectal cancer therapy [235]. It also inhibits cancer cell migration in renal cell carcinoma by suppressing HOTAIR, stopping the cells from spreading [236]. In prostate cancer cells, resveratrol boosts the cancer suppressor lncRNA (PCAT29), suppressing tumor growth by preventing metastasis [237]. It showed potential in treating lung and colorectal cancer by targeting specific genes. Epigallocatechin gallate (EGCG), a phenolic compound, restricts cancer cell growth by inducing apoptosis and targeting key lncRNAs in lung and ovarian cancer cells, dysregulating energy metabolism and fatty acid synthesis [238,239]. EGCG and Dox had a synergistic impact on inhibiting osteosarcoma cell proliferation that involves targeting LncRNA SOX2OT variation 7. It is also reported to have downregulated lncRNA-AF085935 in hepatocellular carcinoma cells and suppressed tumor progression. The exact mechanism is still yet to be deciphered [240]. Gamboic acid (GA), a xanthonoid derived from garcinia resin, triggers apoptosis by suppressing EZH2 and enhances tumor suppression and decreases cancer cell viability in bladder cancer cells [241]. Berberine has been demonstrated to treat the damage of non-alcoholic fatty liver disease (NAFLD) by reversing the abnormal liver disorder. It modulates liver metabolism and replenishes the level of Nrf2 and lncRNAs (MRAK052686), which decreases NAFLD [242]. Bharangin, a diterpenoid, induces the expression of tumor suppressor lncRNAs (MEG-3 and GAS-5) and suppresses oncogenic lncRNA H19 expression in breast cancer cells, promoting cell death and apoptosis. It simultaneously suppresses NF-κB activity decreasing cell migration, proliferation, and arresting cell cycle [243].

Various reports are available that demonstrate the ability of phytochemicals to influence miRNA levels. For instance, the resveratrol treatment of breast cancer cells reduced the expression of miR-200c which induced apoptosis in the cells [244]. EGCG also had similar effects in multiple myeloma cells [245]. EGCG was also reported to modulate levels of miR-21 in breast cancer cells controlling NF-kB [246]. It is observed that, in human monocytic leukemia, resveratrol modulates miR-155 expression to reduce ROS levels and inhibit inflammation-driven carcinogenesis. This is achieved by upregulating miR-663 which targets *JunB* and *JunD* [247].

Hargraves et al. [248] demonstrated that artemisinin and artesunate increased miR-34a expression in a dose-dependent manner, leading to the downregulation of CDK4, a key miR-34a target gene, suppressing tumorigenesis in breast cancer cells. Curcumin is also reported to enhance the Nrf2 signaling pathway via miR-153b, miR-200a, and miR-29 [249]. Similarly, it regulated miR-21 expression to inhibit invasion and metastasis in colorectal cancer [250].

Though not much, the effect of these phytochemicals on the modulation of circRNAs has also been explored. Quercetin, which is a prominent antioxidant and free radical scavenger, has been found to target oncogenic circRNAs involved in the PI3K/AKT/mTOR pathway, suppressing tumorigenesis [251]. Similarly, berberine, celastrol, cinnamon aldehyde, curcumin, and other phytochemicals have been explored to target circRNA. For instance, berberine have been observed to modulate hundreds of circRNA including circRNA2499, hsa_circ_0003423, and hsa_circ_0006702 in gastric cancer cells [252]. Celastrol was observed in suppressing circSATB2 which in turn influenced cell invasion, migration, apoptosis, autophagy, and proliferation [141]. Similarly, in non-small cell lung cancer, cinnamon aldehyde can trigger cell death via apoptosis through a new circular RNA call hsa_circ_0043256 [253].

Thus, the above discussion highlights the significant role of phytochemicals in modulating ncRNAs, thereby influencing key molecular pathways involved in oxidative stress and cancer progression. Table 2 provides a quick mechanistic insight into the role phytochemicals in modulating ncRNA. Further research is essential to fully elucidate their ncRNA-modulating potential which is paving the way for novel cancer treatment strategies.

### 7.6. Clinical Significance (In Vitro and In Vivo Studies) of Phytochemicals

Several studies have shown that antioxidants could be used effectively as chemo-preventive and efficient cell proliferation inhibitors, encouraging cell apoptosis and inhibitors of cell proliferation and detoxification enzymes. Therefore, several researchers work with various forms of natural and dietary antioxidants to identify those with the most remarkable ability to prevent cancer growth both in vivo and in vitro, as such compounds have high efficacy for use in disease treatment as well as chemo-protective solid agents. A list of phytochemicals patented for their anticancer effect has been provided in Table 3.

Phytochemicals in the light of chemoprevention have been studied using suitable skin carcinogenic in vitro and preclinical models and has been proven instrumental for skin cancer prevention [289]. However, these orally active flavonoids have been cross-examined for their significant antitumor effects in various preclinical systems of carcinomas, including skin [290]. Numerous tumors create ROS, and the inclusion of ROS in tumor advancement is bolstered by both in vitro and in vivo examinations. ROS likewise played a noteworthy role in the advancement phase of carcinogenesis. Nutrients and phenolic phytochemicals were amongst vegetables’ most predominant cancer prevention agents and were considered to forestall ROS-mediated cancer. Both in vivo and in vitro systems [291] have demonstrated the ability of certain phytochemicals to treat cancer. The remedial effects of phytochemicals on cancerous cells have not yet been explained. Many mechanisms were provided for the phytochemical’s cytotoxicity, including topoisomerase inhibition, kinase, and pro-oxidant actions [292], while several act as antioxidants and scavenge free radicals. However, the flavonoid’s double protective–destructive action has not been precisely understood.

Their pro-oxidant action is probably accountable for their selective anti-proliferative activity. ROS are essential signaling molecules for cell death modulations [293]. Many phytochemicals have pro-oxidant action, mainly in the presence of metal ions like copper [294]. A possible anticancer mechanism has been suggested for the pro-oxidant action of every dietary compound and their capacity to cause mitochondrial dysfunction and, therefore, apoptosis [185]. There appears to be sufficient proof to maintain ROS development mediated by phytochemicals, a pro-oxidant activity, which causes them to prompt apoptosis in cancerous cells. It has been shown that the build-up of H_2_O_2_ is essential to both in vivo and in vitro death of cancer cells caused by Paclitaxel [295]. H_2_O_2_ can result in the selective destruction of cancerous cells due to Paclitaxel-induced reactive species; H_2_O_2_ is vital in facilitating selective anticancer effects [296]. Various studies reported that plant compounds like Daucosterol, Phisapubesin B, catechins, and Hesperetin can induce cell death in animal models and cancer cells [159]. Further, the apoptotic, cytotoxic, DNA-damaging, and ROS-generating activities of naringenin on normal as well as cancer cells in vitro have been researched [178].

## 8. Conclusions and Future Direction

Various kinds of medicinal plants were known as possible cancer modifiers. They were considered as breakthroughs for the prevention and therapy of cancer. It may be said that imperfection of the oxidation cascade may be checked by irreversible cell injury, which can lead to cell death. OS is caused by the imbalance between ROS and antioxidants and is involved in various metabolic as well as degenerative diseases, such as arthritis, hyperglycemia, Alzheimer’s disease, cancer, and Parkinson’s disease. Cancer is a multi-stage cycle involving three distinctive interconnected phases, i.e., tumor initiation, cancer promotion, and its advancement. Plant antioxidants have been shown to have significant potential in the fight against chronic diseases and cancer resulting from free radical-induced OS. Research has reported that bioactive and healthy phenols, primarily flavonoids, have antioxidant properties from certain medicinal plants and display antibacterial, antitumor, anti-mutagenic, anti-carcinogenic, anti-inflammatory, and antiviral properties. Phytochemicals extracted from different spices and herbs, including flavonoids, polyphenols, tannins, terpenoids, flavonols, etc., operate at various phases of tumor growth, inhibit carcinogenesis initiation and promotion, induction of cell differentiation and cell apoptosis in tumors, and inhibit angiogenesis of tumors. Natural antioxidants are reducing agents, hydrogen donors, and singlet oxygen quenchers, slowing oxidation cascades by eliminating the free radical by-products. Hence, inactivation and activation of transcription factors are the primary mechanisms for treating cancer cells by applying medicinal plants to control inflammatory factors. Subsequently, signaling pathways are modulated by essential cell growth and proliferating cascades, and the antioxidants promote cell cycle arrest and cancer cell death. However, due to limited bioavailability and solubility, clinical trials of most of the phytochemicals have not shown any particular success.

## Figures and Tables

**Table 1:** Some prominent phytochemicals against cancer and their modes of action.

Compound Name	Structure (PubChem)	Function	Target Cells	Modes of Action	Ref.
Oridonin Or Rubesecensin A	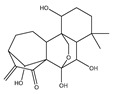	Anti-proliferative	HeLa	Induction of oxidative stress via targeting thioredoxin reductase (TrxR).	
		Apoptotic	HepG2	Oxidative stress induction via mitochondrial signaling pathway.	[91]
		Protection against arsenic-induced toxicity	UROtsa	Activation of NrF-2-mediated response, reduced formation of reactive oxygen species (ROS), and improved cell survival after arsenic challenge.	[92]
Costunolide	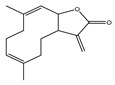	Antioxidant effect	MCI-7 and MDA-MB-231	Decrease TBARs level and increase in SOD, catalase, and GPx.	[93]
Magnolol	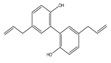	Antioxidant	3T3-L1	Increase in SIRT-1 and decrease in ROS and FAS levels.	[94]
				Oxidative stress-induced apoptosis.	[95]
		Anticancer	Skin cancer in SKH-1 mice and A431	Go/G1 arrest and decrease in levels of CyclinD1 and Cyclin A.	[96]
			U373 CCA	Inhibition of NF-κB pathway and decrease in Ki67, MMP-2, MMP7, and MMP9 levels.	[97]
Pseudolaric Acid	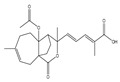	ferroptosis	Glioma cells	Increase in ferrous ion levels and H_2_O_2_ levels, lipid peroxidation, GSH depletion, and Nox$ activation.	[98]
Isoalantolactone	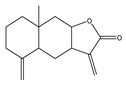	Anticancer	DU-145 and PC-3	Induction of oxidative stress, increase in ROS, and activation of JNK pathway.	[99]
		Apoptosis	HeLa	Oxidative stress by TrxR inhibition and increase in ROS.	[100]
Jaceosidin	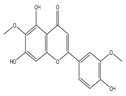	Apoptosis	MCF10A-RAS	Increase in ROS and Bax and decrease in Bcl-2 levels. Inhibition of ERK1/2 activation.	[101]
			U87	G2/M arrest, upregulation of p53 and Bax, and release of cyt-*c* and activation of caspase 3.	[102]
Caticin Or Casticine Or Vitexicarpin	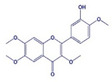	Anticancer	HeLa	G2/M arrest and antimitotic activity.	[103]
			K562 and A2780	Downregulation of cyclin B and activation of p21.	[104]
			HCT-15	Mitotic arrest via PI3k/Akt pathway.	[105]
		Apoptosis	HT-29, HCT-116, SW480, and CaCo-2	Increase in caspase 3.	[106]
			B16F10	Increase in ROS and ASK1/JNK/Bim signaling cascade.	[107]
				Induction of DNA damage via inhibition of DNA repair proteins and decrease in MGMT and MDC1 levels.	[108]
Evodiamine	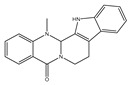	Anticancer	HT-29 and HCT-116	Inhibition of migration of invasion via increase in SIRT-1 and decrease in MMP-9 and acetyl NR-kappaBp65.	[109]
		Apoptosis	A375-S2	Via PI3K/Akt/caspase and FasL/NF-κB signaling.	[110]
Parthenolide	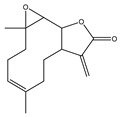	Anticancer	MDA-MB23, BT-20, and MDA-MB43	Oxidative stress, mitochondrial dysfunction, and necrosis.	[111]
			HeLa	TrxR1- and TrxR2-mediated increase in ROS.	[112]
Rhein Or Cassic acid	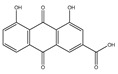	Anticancer	A549, PC9, and PC9	Inhibition of proliferation and migration via Stat3/Snail/MMP2/MMP9 pathway.	[113]
			A2780 and OV2008	Inhibition of migration through downregulation of MMP.	[114]
		Apoptosis	HL-60	ROS-independent mitochondrial death pathway.	[115]
Resveratrol	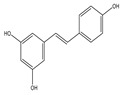	Anticancer	HeLa and MDA-MB-231	Increase in ROS production and decrease in SOD activity and GSH levels.	[116]
		Apoptosis	A375SM	Induction of ROS generation and ER stress and cell cycle arrest. Downregulation of Bcl-2 expression and upregulation Bax.	[117]
Curcumin	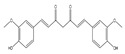	Anti-proliferative	MCF7, HCT116, and A549	Modulation of oxidative stress, regulation of fibrosis, SIRT1 activation, and induction of cellular apoptosis.	[118]
		Antitumor	Colorectal cancer and CT-26 cell	Suppression of angiogenesis and cell proliferation and induction of oxidative stress.	[119]
EGCG	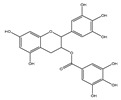	Anti-proliferative	CAL27, HSC-2, and HSG1	Pro-oxidant effect by potentiation of Fe^2+^-induced lipid peroxidation.	[120]
Esculetin	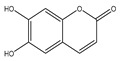	Anti-proliferative	Hep-2, TU-212, and M4e	Inhibition of Janus Kinas (JAK)-signal transducer and activator of transcription-3 (STAT3) activation. Cell cycle arrest at G1/S phase.	[121]
		Apoptosis	PANC-1, MIA PaCa-2, and AsPC-1	Loss of Nrf2-KEAP1 interaction by binding of esculetin with KEAP1 directly.	[122]
Genistein	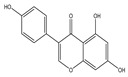	Anticancer	MCF-7	Modulation of oxidative stress according to ERα/ERβ ratioG2/M arrest, increased H2O2, and production of filopodia.	[123]
			HT29 and SW620	Expression of inflammation-related genes increased. NF-kB translocation to the nucleus was increased.	[124]
Hesperidin	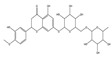	Anti-proliferative	Prostate cancer cells, PC3, and DU145	Generation of ROS and induction of mitochondrial membrane depolarization and endoplasmic reticulum stress.	[125]
		Apoptosis	HepG2	Induction of mitochondrial pathway and death receptor pathway.	[126]
Quercetin	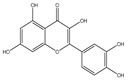	Antiproliferation	MDA-MB-231, MDA-MB-468, and MCF cells	Nrf2-dependent oxidative stress.	[127]
Rosmarinic Acid	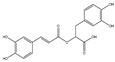	Antioxidant, anti-inflammatory, and anti-metastasis	A549	Modulation of c-Jun, NF-κB, and Akt signaling pathways.	[128]
Carvacrol	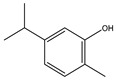	Antioxidant effects and apoptosis	Gastric carcinoma in Wistar rats	Induction of oxidative stress.	[129]
Luteolin	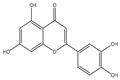	Apoptosis	HT-29	Upregulation of Bax, downregulation of Bcl-2, activation of caspase-9, and caspase-3.	[130]
			SNU-407	Upregulation of Nrf2 expression by DNA demethylase and the interaction of Nrf2 with p53.	[130]
			Bladder cancer cell line and T24	Inhibition of cell survival and induction of G2/M cell cycle arrest, p21 upregulation, and downregulation of phospho(p)-S6, via mTOR signaling. Upregulation of TRX1 and reduction in intracellular ROS production.	[131]
Tannic acid	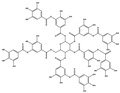	Apoptosis	Prostate cancer cell, C4-2, DU145, and PC-3	Induction of ER stress by ROS. Inhibition of lipogenic signaling and suppression of lipid metabolic pathways. Downregulation of proteins responsible for lipogenesis.	[132]
Berberine	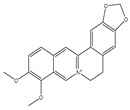	Apoptosis	MCF-7 and MDA-MB-231 cells	Increased production of ROS with activation of the pro-apoptotic JNK signaling.	[133]
			Ovarian cancer cells	Induction of oxidative DNA damage and impairment of homologous recombination repair combined increases sensitivity to PARP inhibition.	[134]
Thymoquinone	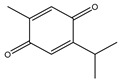	Antitumor and apoptosis	NSCLC	Generation of ROS.	[135]
Thymol	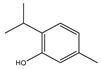	Anticancer and apoptosis	T24 and SW780	Generation of ROS.	[136]
Phytic acid	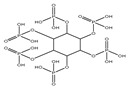	Anticancer	Colon cancers	Increased ROS.	[137]
Chrysin	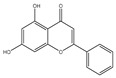	Anticancer and apoptosis	Prostate cancers	Inactivation of the ROS-mediated Akt/mTOR pathway.	[138,139]

**Table 2:** Mechanistic insights into the modulation of ncRNAs by phytochemicals in the regulation of carcinogenesis.

Phytochemical	Target ncRNA Type	ncRNA Example(s)	Mechanism/Effect	Cancer/Condition	References
Curcumin	lncRNA	↓ H19 and ↓ ROR	Suppresses Wnt/β-catenin pathway.	Various cancer types	[110,233,234]
		↓ HOTAIR	Inhibits cancer cell migration and induces growth arrest.	Renal cell carcinoma	[236]
	miRNA	↓ miR-153b, ↑ miR-200a, and ↑ miR-29	Enhances Nrf2 pathway and provides hepatoprotection.	Liver cells	[249]
		↓ miR-21	Induced the expression of the tumour suppressor PDCD4 and inhibited invasion and metastasis.	Colorectal cancer	[250]
	circRNA	↓ circSATB2 and ↓ circFOXM1	Modulates invasion, migration, and apoptosis.	Diverse cancers	[141]
Resveratrol	lncRNA	↑ PCAT29	Boosts tumor suppressor lncRNA and the inhibition of metastasis.	Prostate cancer cell	[237]
	miRNA	↓ miR-200c	Induces apoptosis.	Breast cancer	[244]
		↓ miR-155 and ↑ miR-663	Targets *JunB* and *JunD*, reduces ROS, and inhibits inflammation-driven carcinogenesis.	Human monocytic leukemia	[247]
EGCG (Green Tea)	lncRNA	Modulation of multiple lncRNA	Induces apoptosis and disrupts energy metabolism.	Lung and ovarian cancer	[238,239]
		↓ SOX2OT variation 7	EGCG reduced Dox-induced pro-survival autophagy. Decreased the stemness and abated the drug resistance in osteosarcoma cells and deactivated the Notch3/DLL3 signaling cascade.	Osteosarcoma	[240]
		↓ AF085935	Suppress cell proliferation.	Hepatocellular carcinoma	[240]
	miRNA	↓ miR-21	Modulates NF-κB pathway.	Breast cancer	[246]
		↑ miR-200c	Induces apoptosis.	Breast cancer	[245]
		↓ miR-200c	Suppressing Notch and Bmi1, Ezh2, and Suz12.	Colorectal cancer	[254]
Gamboic Acid (GA)	lncRNA	↓ EZH2	Induces apoptosis.	Bladder cancer	[241]
Bharangin	lncRNA	↑ MEG-3, ↓ GAS-5, and ↓ H19	Inhibition of NF-κB and induction of apoptosis and cycle arrest.	Breast cancer	[243]
Artemisinin/Artesunate	miRNA	↑ miR-34a	Increases expression and downregulates CDK4.	Breast cancer	[248]
Quercetin	circRNA	↓ PI3K/AKT/mTOR-related circRNAs	Suppresses tumorigenesis by targeting oncogenic circRNAs.	Diverse cancers	[251]
Berberine	lncRNA	↑ MRAK052686	Restores Nrf2/lncRNA levels.	NAFLD and liver dysfunction	[242]
		↓ HOTAIR	Induction of metastasis.	Lung cancer	[255]
	miRNA	↑ miR-34a-5p	Suppression of *KRAS* and *c-MYC* to prevent and reverse tumorigenesis.		
	circRNA	Diverse circRNA	Affects cell proliferation via Notch, MAPK, and NF-*κ*B signaling pathways.	Gastric cancer	[252]
Cinnamon Aldehyde	circRNA	↑ hsa_circ_0043256	Modulates apoptosis, autophagy, and proliferation.	Diverse cancers	[253]
Celastrol	circRNA	↓ circSATB2	Inhibiting miR-33a-5p/E2F7 axis.	Lung cancer	[205]

Note: ↑ represents upregulation, and ↓ represents downregulation.

**Table 3:** Patents on phytochemicals having therapeutic properties that induce oxidative stress to prevent cancer (since 2011 onwards).

Year	Patent Number	Title	Applicants	Ref.
2012	US9078891B2	7-Hydroxychromones as potent antioxidants.	Unigen Inc. Tacoma, WA, USA	[256]
2012	WO2012021981A1	Novel phytochemicals from extracts of maple syrups and maple trees and uses thereof.	Fédération Des Producteurs Acéricoles Du Québec, University Of Rhode Island, KGN, USA	[257]
2012	WO2012017451A1	A bio-stabilized resveratrol formulation.	Sanjeev Khandelwal	[258]
2012	US20120045532A1	Anticancer methods employing extracts of *Gleditsiasinensis* Lam.	Bionovo Inc., Emeryville, CA, USA	[259]
2013	WO2013148682A1	Methods and compositions for controlled delivery of phytochemical agents.	University of Louisville Research Foundation ULRF	[260]
2013	US20130310332A1	Maple tree-derived products and uses thereof.	Federation Des Producteurs Acericoles Du Quebec Rhode Island University, KGN, USA	[261]
2013	KR101219520B1	Anti-inflammatory, anti-oxidative, or antibacterial compositions.	Michel Bio Co., Ltd., Dallas, TX, USA	[262]
2014	US8734859B1	Molecular combinations for cancer or other disease treatment.	Sirbal Ltd., Limassol, Cyprus	[263]
2014	KR101450480B1	Pharmaceutical composition having antioxidants comprising chlorophylls from isolated soybean.	Kangwon National University Industry-University Cooperation Foundation	[264]
2014	US8858995B2	Methods and compositions for controlled delivery of phytochemical agents.	University of Louisville Research Foundation ULRF	[265]
2014	US20140127179A1	Natural killer cell formulations.	Scientific Formulations LLC, Allentown, PA, USA	[266]
2014	US20140088052A1	Chalcone derivatives as nrf2 activators.	United States, Health and Human Services C/O Office of Technology Transfer National Institutes Of Health, Secretary of, Department of Johns Hopkins University	[267]
2014	US20120122943A1	Melampomagnolide B derivatives as antileukemic and cytotoxic agents.	University of Kentucky Research Foundation University of Rochester	[268]
2015	US9187397B2	Therapeutic curcumin derivatives.	STC UNM	[269]
2015	US20140141082A1	Compositions containing enriched natural crocin and/or crocetin and their therapeutic or nutraceutical uses.	Song Gao	[270]
2015	US20150216918A1	Fermented soy nutritional supplements including mushroom components.	Vijaya Nair	[271]
2015	US9180155B2	Compositions from *Nigella Sativa*.	Bio Nexus Ltd., Netanya, Israel	[272]
2015	CN104623670A	Compositions containing enriched natural crocin and/or crocetin and their therapeutic or nutraceutical uses.	Takamatsu	[273]
2016	US9295698B2	Krill oil and carotenoid composition and its associated method and delivery system.	US Nutraceuticals LLC, Eustis, FL, USA	[274]
2017	US9849153B2	Compositions and methods for preventing and treating diseases and environmentally induced health disorders.	Royal Institution for the Advancement of Learning	[275]
2017	EP2068864B1	Therapeutic uses of urolithins.	University of California	[276]
2018	US9920063B2	Melampomagnolide B derivatives.	BioVentures LLC University of Colorado	[277]
2019	CN110538301A	Radix tetrastigme compound composition for enhancing antitumor and antioxidant activities and preparation method thereof.	Nanchang University	[278]
2019	CA2832273C	Pharmaceutical compositions for oral administration, comprising a tomato oleoresin.	Lycored Ltd., Beer Sheva, Israel	[279]
2019	US20190275119A1	Pro-oxidant cancer chemo-suppressors and chemo-protectors and methods of use related thereto.	Randolph M Howes	[280]
2019	WO2019155337A1	Compositions comprising a cannabinoid and punicalagin and methods of use thereof.	Scicann Therapeutics Inc., Dr. Mark Friedman Ltd., Toronto, ON, Canada	[281]
2021	US11026982B2	Method for reducing the likelihood of developing bladder or colorectal cancer in an individual human being.	Seed Health Inc., Venice, CA, USA	[282]
2021	US20160331707A1	Antioxidant compositions for treatment of inflammation or oxidative damage.	Perio Sciences LLC, Rockford, IL, USA	[283]
2021	US10967025B2	Herbal nutraceutical formulation to reduce oxidative stress, viral and microbial infections, and inflammation.	Moringo Organics Inc., Vancouver, BC, Canada	[284]
2021	US10912758B2	Compositions with ketogenic agents, cannabinoids, plant-derived substances, and micronutrients.	Robert Firger, Gerald M. Haase	[285]
2022	US20220257640A1	Nutritional formulation for cancer prevention.	Individual	[286]
2022	US11433134B2	Aqueous formulation of erythropoiesis-stimulating protein stabilized by antioxidants for parenteral administration.	Amgen Inc., Oaks, PA, USA	[287]
2022	US20180250264A1	Compositions for improved nrf2 activation and methods of their use.	Pathways Bioscience LLC, Aurora, CO, USA	[288]

## Data Availability

The information supporting this study’s findings is available in this article.

## Abbreviations

OS	Oxidative stress
ATP	Adenosine triphosphate
ROS	Reactive oxygen species
CAT	Catalase
SOD	Superoxide dismutase
PI3K	Phosphatidylinositol 3-kinase
mTOR	Mechanistic target of rapamycin
NF-κB	Nuclear factor κB
MAPK	Mitogen-activated protein kinase
ERK1/2	Extracellular signal-regulated kinase1/2
JNK	c-Jun N-terminal kinase
STAT3	Signal transducer and activator of transcription-3
EGCG	EpigalloCatechin-3-gallate
